# A specificity map for HTLV-1 Tax/PDZ interactions

**DOI:** 10.1186/1742-4690-8-S1-A162

**Published:** 2011-06-06

**Authors:** Karim Blibek, Jean-Claude Twizere

**Affiliations:** 1 Cellular and molecular biology, Univesity of Liège, Gembloux Agro Bio-Tech, 5030, Belgium; 2 Immunology and virology, GIGA Research Center, Univesity of Liège, Liège, 4000, Belgium

## 

Human T-cell leukemia virus type I (HTLV-1) encodes a Tax oncoprotein that is critical for both viral replication and cellular transformation. HTLV-1 Tax possesses a PDZ domain binding motif (PBM) at its C-terminus that is essential for its transforming activity in a Rat-1 model and for IL-2. Tax has been shown to interact with several PDZ domain containing proteins including PSD-95, Beta1-syntrophin, the precursor of interleukin-16, the mammalian homolog of the Drosophila discs large tumor suppressor protein Dlg, PDLIM2, Lin7, hTid1, Tip1, hScrib and MAGI3. In the 15th International Conference on Human Retrovirology: HTLV and Related Retroviruses, we will present a specificity map for the Tax/PDZ domain interactions generated using the human ORFeome 5.1. and we will focus on some of the new Tax/PDZ interactions.

